# Targeting chondroitinase ABC to axons enhances the ability of chondroitinase to promote neurite outgrowth and sprouting

**DOI:** 10.1371/journal.pone.0221851

**Published:** 2020-01-21

**Authors:** Priscilla Day, Nuno Alves, Esther Daniell, Debayan Dasgupta, Rosalie Ogborne, Ashley Steeper, Mansoor Raza, Clare Ellis, James Fawcett, Roger Keynes, Elizabeth Muir

**Affiliations:** 1 Department of Physiology Development and Neuroscience, University of Cambridge, Cambridge, United Kingdom; 2 John Van Geest Centre for Brain Repair, University of Cambridge, Cambridge, United Kingdom; University of Toronto, CANADA

## Abstract

**Background:**

There is currently no effective treatment for promoting regeneration of injured nerves in patients who have sustained injury to the central nervous system such as spinal cord injury. Chondroitinase ABC is an enzyme, which promotes neurite outgrowth and regeneration. It has shown considerable promise as a therapy for these conditions. The aim of the study is to determine if targeting chondroitinase ABC expression to the neuronal axon can further enhance its ability to promote axon outgrowth. Long-distance axon regeneration has not yet been achieved, and would be a significant step in attaining functional recovery following spinal cord injury.

**Methodology/Principal findings:**

To investigate this, neuronal cultures were transfected with constructs encoding axon-targeted chondroitinase, non-targeted chondroitinase or GFP, and the effects on neuron outgrowth and sprouting determined on substrates either permissive or inhibitory to neuron regeneration. The mechanisms underlying the observed effects were also explored. Targeting chondroitinase to the neuronal axon markedly enhances its ability to promote neurite outgrowth. The increase in neurite length is associated with an upregulation of β-integrin staining at the axonal cell surface. Staining for phosphofocal adhesion kinase, is also increased, indicating that the β-integrins are in an activated state. Expression of chondroitinase within the neurons also resulted in a decrease in expression of PTEN and RhoA, molecules which present a block to neurite outgrowth, thus identifying two of the pathways by which ChABC promotes neurite outgrowth.

**Conclusions / Significance:**

The novel finding that targeting ChABC to the axon significantly enhances its ability to promote neurite extension, suggests that this may be an effective way of promoting long-distance axon regeneration following spinal cord injury. It could also potentially improve its efficacy in the treatment of other pathologies, where it has been shown to promote recovery, such as myocardial infarction, stroke and Parkinson’s disease.

## Introduction

There is currently no effective treatment for promoting regeneration of injured nerves in patients following brain trauma or spinal cord injury (SCI). The principal cause of disability that results from such injuries is the regenerative failure of mammalian CNS axons. This is due in part to upregulation of growth-inhibitory chondroitin sulphate proteoglycans (CSPGs) in the region of injury [1, 2,3].

Chondroitinase ABC (ChABC), an enzyme isolated from the bacterium *P*. *Vulgaris*, promotes axon regeneration following CNS injury. It functions by removing growth-inhibitory CSPGs at the lesion site and this also promotes neural plasticity by dissolution of perineuronal nets [4, 5,6]. This latter action results in the formation of new synaptic connections by intact, undamaged neurons, with the beneficial consequence of allowing spared axons to replace the function of damaged neurons. This is of particular importance for promoting recovery following SCI, as most injuries are not complete and thus spared axons remain.

The robustness of efficacy of ChABC in experimental SCI has been demonstrated in many injury models and in several mammalian species [4, 5, 7]. Critically, it is also effective in cat, mouse and rat models of chronic SCI, thus greatly extending the number of patients who could potentially benefit from this treatment [7, 8, 9]. This makes it a very strong candidate for treatment of human SCI. Moreover, ChABC also has the potential for wider therapeutic application, since it has recently been shown to improve outcome following peripheral nerve injury [10], and to promote cardiac sympathetic nerve regeneration following experimental myocardial infarction [11]. Additionally, there are an increasing number of studies describing beneficial results of the enzyme in experimental models of stroke [12, 13]. However, the large size of the enzyme hinders its diffusion from the site of injection (intrathecal) to the lesion site. It is also unstable at body temperature, thus multiple applications are required for efficacy. This increases the risk of causing further trauma and infection. These drawbacks could be circumvented by a gene therapy approach of enzyme delivery, but when the bacterial gene is introduced into mammalian cells, they do not secrete active enzyme. We identified the changes to the bacterial gene that are required to allow secretion of this bacterial enzyme by mammalian cells, thus making a gene therapy approach to treatment possible [14]. Delivery of the enzyme by a viral vector results in marked enhanced efficacy, in a rat model of SCI. Large scale digestion of CSPGs in and around the lesion site occurs, which is accompanied by behavioural improvements (ladder walk test). There is also a large reduction in cavity size of the lesion, which can be attributed to the newly identified immunomodulatory properties of the enzyme [15]. These findings are very encouraging and exceeded expectation. However, for efficacy in humans long-distance axon regeneration will be required, and this has not yet been achieved. We have therefore modified the gene further, targeting the enzyme to axons to determine if this can further enhance neurite outgrowth. We show here that such targeting not only markedly enhances the enzyme’s ability to promote neurite outgrowth, but also promotes sprouting. This is an important finding, as this is an important mechanism for promoting recovery following SCI.

We also unveil some of the previously unidentified mechanisms underlying these effects. We used an *in vitro* model of neurite outgrowth to study the effects of targeting ChABC to the axonal compartment of neurons and to investigate the signalling pathways involved. SH-SY5Y cells are used as a model of neurons and chondroitin-4-sulphate (CSA) as a substrate inhibitory to neurite outgrowth. SH-SY5Y cells are a human neuronal cell line, derived from the sympathetic nervous system. They differentiate into neurons following treatment with retinoic acid [16]. Critically, these cells have properties in common with cortical neurons, one of the neuronal subtypes we particularly wish to target, because their regeneration is important for recovery following SCI. In common with cortical neurons, SH-SY5Y cells extend short neurites, which are refractory to most treatments designed to promote neurite outgrowth. Moreover, we show here, that also in common with cortical neurons, they produce CSPGs. We have also noted that results we have obtained with this cell line are recapitulated in other neuronal cell lines and primary cultures of neurons, suggesting that are useful for predicting general neuronal behaviour.

## Materials and methods

### Construction of an expression plasmid encoding ChABC with an axon-targeting signal

The eukaryotic expression plasmid, pcDNA 3.1-(Invitrogen), encoding mammalian compatible ChABC [14] and containing a mutated stop codon, was cut with EcoRI/BamHI. This was ligated to an oligonucleotide coding for an axon-targeting sequence from amyloid precursor protein intracellular domain [17]. The oligonucleotide also incorporated a tetracysteine motif. The bi-arsenical labelling reagents FIAsH-EDTA2 and ReAsH-EDTA2, become fluorescent when they bind to recombinant proteins containing this motif. This provides a sensitive method of determining the subcellular localisation of proteins using fluorescence microscopy. The tetracysteine motif reportedly rarely occurs in endogenous proteins, so incorporating the sequence into target proteins provides a highly specific target for protein labelling.

Oligonucleotide sequences.

                    N CCPGCC

  5’AATTC CTG AAT TGC TGC CCC GGC TGC TGC ATG GAG CCC ATG

  EcoRI          Tetracysteine tag

    Y  E  N  N  P  T  Y  K  F  F  E  Q  M  Q  N

  GGA TAT GAG AAT CCA ACT TAC AAG TTC TTT GAG CAA ATG CAG AAC

                              Axon targeting signal

 TAG G 3’

  stop BamHI

  5’GATCC CTA GTT CTG CAT TTG CTC AAA GAA CTT GTA AGT TGG ATT

  BamH1

  CTC ATA TCC CAT GGG CTC CAT GCA GCA GCC GGG GCA GCA ATT CAG G 3’

                                                                                                                                          EcoR1

### Cell culture

SH-SY5Y Neurons: (ATCC CRL2266^TM^). These were propagated in DMEM, 10% FCS 100 units/ml penicillin and 100μg/ml streptomycin. Cells were passaged 24h prior to transfection and plated to achieve 60% confluency on the day of transfection. Dorsal Root Ganglion neurons (DRGs): Adult Sprague-Dawley rats, 250-400g, were supplied by Charles River Laboratories, Margate, UK. They were housed under a 12h light/dark cycle with *ad libitum* access to food and water. Euthanasia was carried out by decapitation. This is considered a regulated procedure under the Animals Scientific Procedures Act 1986 and as such was authorised under a project License (70/7920, 19b6 “Acquisition of Tissue”) which was ethically reviewed by the University of Cambridge Animal Welfare &Ethical Review Body (AWERB) prior to submission and subsequent approval by the Secretary of State. The University of Cambridge holds an Establishment License (80/2802, X81BD37B1) and is committed to animal welfare, with all animal facilities designated under the above Act. DRGs were dissected from adult rats, dissociated with 2% collagenase (Sigma) and 0.1% trypsin (Invitrogen) for 15 min at 37°C, then washed Hanks balanced salt solution (HBSS) prior to plating. Cultures were grown in Neurobasal medium (Invitrogen) supplemented with B27 (Invitrogen), glutamic acid, penicillin and streptomycin (Sigma).

### Transfections and transductions

For the neurite outgrowth assays, SH-SY5Y neurons were transfected in serum-free medium with a plasmid encoding non-targeted ChABC or axon-targeted ChABC, using Xfect (Clontech), according to the manufacturer’s instructions. GFP-transfected cells or non-transfected cells served as controls. A plasmid encoding mcherry or FIAsH-staining was used to assess transfection efficiency in the groups transfected with the ChABC plasmids. After 5h, the medium was replaced with medium containing serum and the cells incubated overnight. 24h post-transfection they were plated onto 4 well slides at a density of 1.7x10^-5^ cells/well in DMEM, containing 1%FCS and antibiotics. Retinoic acid, 10^-6^M, was added to promote differentiation of these cells into cells with morphological and biochemical characteristics of mature neurons [16]. For the PTEN and RhoA expression experiments, ChABC was introduced into the SH-SY5Y neurons via a lentivirus described in Zhao et al.[5]. This encodes non-targeted ChABC, expressed under the control of a CMV promoter. The cells are split the day before transduction to achieve 50–70% confluence on the day of transduction. Transductions are carried out in DMEM, 10%FCS and polybrene (8μg/ml), (Millipore). 2μl of vector/25cm^2^ flask was added to 1ml of medium and the cells incubated with the vector for 24h. It was then then replaced with fresh medium, consisting of DMEM with10%FCS and antibiotics. Vector concentration was 99μg/ml P24. Expression peaked at 48h, and remained stable for at least 10days, as determined by the Morgan-Elson enzyme assay. The PTEN experiments and neurite outgrowth assays were conducted during this period.

DRGs were transfected by microporation using a neon kit (Invitrogen), according to the manufacturer’s instructions. Briefly, 0.5μg of plasmid (encoding GFP (control), non-targeted ChABC or axon-targeted ChABC) was added to~1x10^5^ cells suspended in electroporation buffer (invitrogen). These were then electroporated at 1200V, 20ms, 2pulses, then plated onto laminin/CSA coated coverslips at 2.0–4.0x10^6^ cells/cm^2^ in serum free medium (neural basal medium withB27 supplement). Neurite lengths were measured 72h post-plating.

### Substrates for neurite outgrowth

#### SH-SY5Y cells

4 well slides (Millipore), were coated with 100μg/ml poly-l-lysine and incubated at 37°C overnight. The poly-l-lysine was then removed and the slides washed with PBS and left to dry. The poly-l-lysine-coated slides were then coated with 10ug/ml laminin in DMEM. This provides a substrate permissive for neurite outgrowth. Chondroitin-4-sulphate (CSA), is known to be inhibitory to axon outgrowth [18]. CSA from bovine trachea was used, which is an alternating copoly β-glucuronic acid-(1–3)-Nacetyl-β-galactosamine-4-sulphate-(1–4).To determine the concentration of CSA required to inhibit neurite outgrowth, slides were coated with a laminin/CSA mixture containing a constant amount of laminin and CSA concentrations between 10ng/ml and 75ug/ml. Non-transfected cells were plated onto these slides and immuno-stained for the neuronal marker β3-tubulin to visualise the neurites [19]. Neurite lengths were measured, and statistically compared to those obtained on laminin alone. There was a significant difference in neurite length between the laminin and the 75 μg/ml CSA, (MWU test) *** P<0.001). The other concentrations of CSA, (25 and 50 μg/ml) did not show any difference in neurite outgrowth compared to the laminin control, S1 Fig. Therefore, to produce an inhibitory environment for the neurite outgrowth assays, poly lysine-coated slides were coated with a mixture of 75ug/ml CSA and 10μg/ml laminin, diluted in DMEM. They were then incubated overnight at 37°C, and washed with DMEM, prior plating the cells.

#### DRGs

Coverslips were coated with laminin 1μg/ml or laminin and CSA, 25μg/ml, a concentration previously shown to be inhibitory to neurite outgrowth of these neurons (three coverslips/group, each experiment was repeated at least three times).

### Immunohistochemistry

Following fixation with 4% paraformaldehyde, cells were permeabilised in 0.2% Triton X-100 for 5 minutes and washed 3 times in phosphate buffered saline (PBS). They were then blocked: first with Image-iT FX Signal Enhancer (Invitrogen) for 30 minutes in a humid environment; then with block buffer (0.3% Triton X-100, 10% goat serum in PBS) for 2 hours. They were then stained with primary antibody, in block buffer overnight at 4°C. Cells were then washed 4 times, 5minutes each in PBS, before a 1h incubation in the secondary antibodies. After three 5minute washes in PBS, they were mounted with Prolong Gold anti-fade reagent (Invitrogen) and coverslipped. The primary antibodies used were: Mouse monoclonal anti-β tubulin III antibody, clone 2G10, (1:1000, Sigma, T8578), immunogen: synthetic peptide corresponding to amino acids 436–450 of human neuronal specific β-tubulin III, was used to visualize the neurites. Staining with anti-β tubulin III also confirmed that the SH-SY5Y cells had successfully differentiated into mature neuron-like cells, as β-tubulin is almost exclusively expressed in neurons [20]. Rabbit polyclonal anti-GFP (1:1000, Invitrogen,#A-11122) immunogen: jellyfish, *Aequorea Victoria*, was used to stain GFP-transfected cells, and mouse monoclonal anti-β1-integrin (1:200,Millipore, MABT1502,) clone, 102DF5, immunogen: tissue extract from human myometrium, was used to stain SH-SY5Ycells or rabbit polyclonal anti-pFAK (phosphorylated focal adhesion kinase)(1:500, Invitrogen, #44–636) immunogen: synthetic peptide, that contains tyrosines 579&580, (which are conserved in rat and human), was used to stain DRGs. Cells stained for β1-integrin, were not permeabilised, in order to restrict detection to surface β-integrin expression. Staining for β1-integrin with DRGs was found to be weak, therefore these cells were stained for the presence and activation (phosphorylation) of focal adhesion kinase (pFAK) instead. This allows a direct assessment of integrin activation. The activation of integrin triggers a signalling cascade within the cell (‘outside-in’ signalling) and integrin clustering results in a rapid auto-phosphorylation of FAK [21]. Intracellular RhoA and PTEN, were detected using anti-RhoA, mouse monoclonal (1:200,Abcam,ab54835), clone 1B12, immunogen: full length recombinant protein, corresponding to amino acids 1–194 of the human protein and rabbit (monoclonal) and anti-PTEN (1:250, cell signalling, mab#9559) immunogen: synthetic peptide corresponding to the carboxy terminus of human PTEN, which detects total PTEN levels. Secondary antibodies were: AlexaFluor488 goat, anti-mouse IgG, (1:2000, Invitrogen), AlexaFluor488 goat anti-rabbit IgG (1:2000, Invitrogen). Detection of CSPGs on the surface of SH-SY5Y cells was carried out by staining of non-permeabilised cells, with mouse monoclonal anti-CS56 (1:250, Invitrogen, #MA-83055) RRID, AB929919, immunogen: ventral membranes of chicken gizzard fibroblasts, which detects intact CSPGs. The secondary antibody used for this reaction was AlexaFluor 488 goat anti-mouse IgM, 1:400, Invitrogen).

### Morgan-Elson enzyme assay

This enzyme assay measures ChABC activity by the N-acetylation of product disaccharides and subsequent reaction to give a coloured product. The reaction contains 100μl of 40mM sodium acetate, 40mM Tris-Cl pH8.0, 10mg/ml chondroitin-6-sulphate (Sigma), mixed with 20μl of enzyme sample. *P*.*Vulgaris* ChABC (Sigma) was used as a standard. The reaction was incubated at 37°C for 20min, then stopped by boiling for 1min. Potassium borate solution (0.8M, pH9.1, 100μl) was added and the mixture boiled for 7min. It was then chilled on ice and centrifuged in a microfuge at 13,000rpm for 10 mins. 1ml of glacial acetic acid was added to the supernatant and mixed before centrifugation for a further 20min. To 1ml of supernatant, 0.4ml of Morgan-Elson Reagent (10g paradimethylamino-benzaldehyde in 100ml of glacial acetic acid containing 12.5% concentrated HCl) was added and incubated at 37°C for 20min. Product was measured by absorbance at 550nm.

### Fluorescence microscopy and image analysis

Images were captured on a Zeiss Axioplan microscope, under red or green fluorescent light (depending on the antibodies used) using a digital camera (QImaging) and QCapture Pro6.1 imaging software. This allows regulation of the acquisition exposures. The exposure, once optimized for photography for any set of photographs, was kept constant for all the photographs in that set.

All image analysis was performed using ImageJ (NIH). A segmented line was used to measure neurite lengths. Measured lengths were converted to their actual size using a multiplication factor, determined from a photograph of a scale bar at 40x power. The average neurite length was then calculated from all neurons on each coverslip, to give a final measurement for each condition. Fluorescent measurements were carried out using a formula adapted by Gavet &Pines [22], as follows:

Whole cell signal is the sum of the intensity of the pixels from one cell.

Axon signal is the sum of intensity of the pixels for the cells' axon.

Background signal is the average signal per pixel for a region selected just beside the cell.

Whole cell or axon signal corrected is the whole cell signal or axon signal with the background signal subtracted.

Cell body signal corrected is the whole cell signal corrected minus the axon signal corrected.

### Data analysis

Data was statistically analysed using IBM SPSS statistics software, (version 21.0; IBM Co, 1 Armonk, NY, USA).

Neurite length and number analysis: The Shapiro-Wilk test was used to test whether the data followed a normal distribution. The data were found not follow a normal distribution, P<0.001. This was the case for both neurite length and number. Therefore, the non-parametric Mann Whitney-U-test (MWU), was used to compare neurite length and number between cells transfected with different constructs and plated onto the different substrates. It was also used to compare cell fluorescence intensities and gene expression analysis. A P value <0.05 was considered significant. Where more comparisons between groups were made, a Holm adjustment was performed. This method enables multiple comparisons to be made between groups statistically analysed by a Mann Whitney-U test. It uses a stepwise approach to compute the significance levels depending on the P value based rank of hypotheses [23]. In the case of the experiments measuring the intracellular levels of PTEN, because our prior interest was in whether neurite length differed between the non-transduced control group and the treatment groups, the primary analysis was a series of three comparison of neurite length of each transduced group with that in the control group, without adjusting for multiple comparisons. Because neurite lengths were highly skewed, the comparison used, was performed on log-transformed data.

### Q PCR: PTEN gene expression

Real time (qPCR) was used to measure intracellular levels of PTEN in the different experimental groups. Differentiated SH-SY5Y cells were resuspended in RNA later (Life Technologies). RNA was extracted using the High Pure RNA Isolation Kit, (Roche), its integrity and concentration was assessed using agarose gel electrophoresis and nanodrop respectively. 1μg RNA was then reverse transcribed into cDNA using Nanoscript Reverse-Transcription Kit (Primer Design), following the manufacturers protocols. Intron-spanning primers were designed, specific for PTEN or ACTB, primer sequences shown in S1 Table.

Real-time quantification was carried out using GoTaq® Probe qPCR Master Mix (Promega, UK) according to manufacturer’s instructions, in a final volume of 10μl. The Light Cycler 480 (Roche, UK) was used for amplification and data acquisition, using the following cycling conditions; 95°C for 10 min, then 40 cycles at 95°C for 15s and 60°C for 1 min. Relative gene expression levels were calculated using the standard curve method, and gene expression was normalised using beta-actin (ACTB) housekeeping gene.

### CSPG detection

Dot blot: Serum-free conditioned medium (DMEM) from SH-SY5Y cells, cultured for 72h, was spotted onto nitrocellulose membrane using a dot blot apparatus. CSPG mix (Millipore) diluted with DMEM were used as a positive control. Half the samples from each group were treated with ChABC for 3h, prior to incubation of the membrane with antibody 2B6 (mouse monoclonal to epitopes exposed by ChABC digestion) 1:500, Seikagaku. This detects a stub epitope exposed by ChABC digestion of CSPGs. The membrane was then washed and incubated with HRP anti-mouse IgG 1:10,000. The signal was detected using chemiluminescence (Luminata) and visualised using hyperfilm (Amersham).

CSPG assay: A Blyscan sulphated glycosaminoglycan assay (Biocolor) [24], was used to detect the presence of glycosaminoglycan chains in the conditioned medium of differentiated SH-SY5Y cells. This dye binding assay is a quantitative measure of intact CSPG GAG chains. A CSPG mix (Millipore) was used as a positive control. Absorbance was measured at 656nm and sulphated-glycosaminoglycan concentrations obtained from a standard curve.

### Rho A experiments

SH-SY5Y neurons were transduced with a lentivirus encoding ChABC (LVC) described in Zhao et al. [5]. Non-transduced cells served as a control. Cells were plated onto slides in DMEM+1%FCS and 10^-6^M retinoic acid to induce differentiation into mature neurons [16]. After 48h, neurons were fixed and immuno-stained for RhoA, followed by staining with an AlexFluor488, secondary antibody, to allow quantitation of RhoA levels by fluorescence measurements.

The axon-targeted ChABC construct was introduced into DRGs via microporation, then plated onto coverslips and stained for Rho A. The presence of intracellular RhoA was detected, using an AlexaFluor 488 secondary antibody.

### PTEN experiments

One group of SH-SY5Y cells were transduced with a lentivirus encoding ChABC (LVC) [5]. A second group were treated with the PTEN inhibitor VO (OH)Pic [25], 100μM(Santa Cruz). A third group were transduced with the lentivirus encoding ChABC and treated with the PTEN inhibitor VO (OH)Pic. Non-transduced cells served as a control. To determine the effect of ChABC and VO(OH)Pic on neurite lengths, cells were plated onto slides coated with CSA, in DMEM+1%FCS and 10^-6^M retinoic acid to induce differentiation into mature neurons [16].The PTEN inhibitor was added to two groups, (PTEN inhibitor alone and PTEN inhibitor + ChABC). After 48h, the cells were fixed and immuno-stained for the neuronal marker β3-tubulin to visualise the neurites. Neurite lengths were measured, and statistically compared to those obtained with control cells. PTEN protein levels were determined by fluorescence measurements. The neurons, prepared as above, were stained with an anti-PTEN antibody followed by an AlexFluor 488 secondary antibody, to allow fluorescence quantitation.

To assess the effect of VO(OH)Pic and ChABC on PTEN gene expression, cells from each group were plated into six, 25cm^2^ flasks and differentiated in the presence of 1%FCS and 10^-6^M retinoic acid. After 48h, RNA was extracted from the cells and qPCR performed, using the PTEN specific primers given in S1 Table. Each experiment repeated three times.

### FIAsH staining

FIAsH staining was adapted from the manufacturer’s instructions (ThermoFischerscientific). The optimal working concentration was determined to be 1.25μg/ml in HBSS with Ca^2+^ and Mg^2+^. Cells were washed x6 with HBSS, then fixed with 4% paraformaldehyde. Cultures were then washed twice with HBSS, permeabilised with 0.2% Triton-X100 in PBS for 5mins, then washed x3 with HBSS. The FIAsH reagent was added (0.5ml/well) and the cultures incubated at RT for 30min, protected from light. BAL buffer was then used to wash the cells (2 washes, 2mins/wash). Once stained, slides were mounted with Prolong Gold anti-fade reagent (Invitrogen), and examined within 48h to avoid fading.

## Results

### Targeting ChABC to the axon promotes neurite outgrowth

Cultures of SH-SY5Y cells transfected with targeted ChABC and plated onto CSA, had significantly longer neurites when compared to cells transfected with GFP, plated onto CSA (MWU-test: P<0.001) (Table 1).

**Table 1:** Median neurite lengths from SH-SY5Y neurons transfected with different constructs and plated onto laminin or CSA.

Construct, substrate	Median neurite length/μm	Z value	Min/Max values	P Value	N
Targeted ChABC laminin	16.2		0.0–200.5		1023
Targeted ChABC CSA	16.8	0.531	0.56–111.4	0.595	1146
GFP,CSA	10.6	13.741	0.0–51.2	<0.0001	465
Targeted ChABC laminin	15.7		0.0–97.44		1005
GFP, laminin	12.3	11.91	0.0–73.36	<0.0001	306

N = number of neurite lengths measured.

Comparison of neurite lengths of cells transfected with targeted ChABC plated on laminin with those plated onto CSA, shows that the inhibitory effect of CSA on neurite outgrowth is reversed, as there is no significant difference in neurite length between the two groups P>0.05, Unexpectedly, cultures expressing targeted ChABC also have longer neurites when plated on laminin, compared to cells transfected with GFP and plated onto laminin (MWU- test P<0.001). The results shown are from one experiment. The experiment was repeated three times.

**Fig 1:**
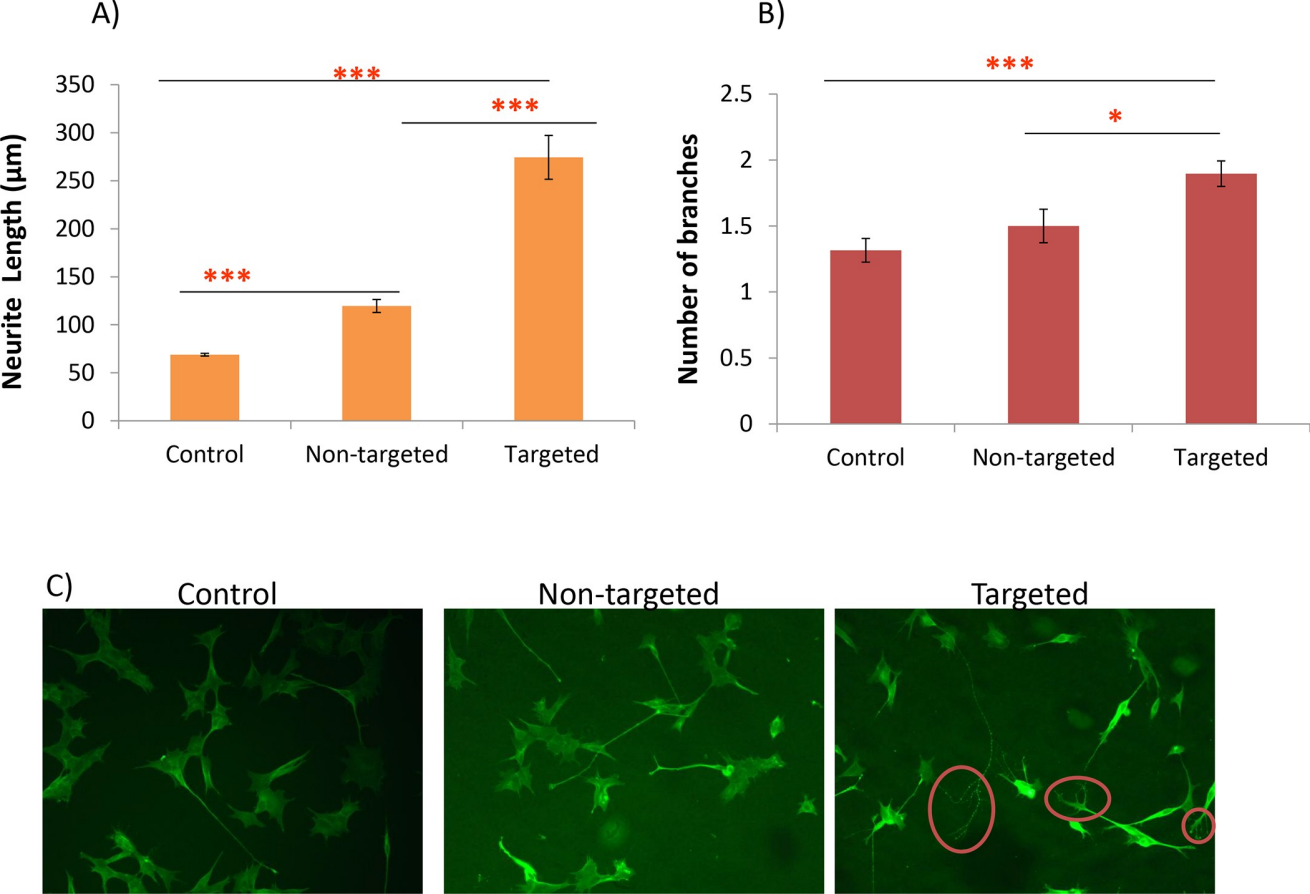
Neurite length and branching of SH-SY5Y neurons transfected with the different ChABC constructs and plated onto CSA. A) SH-SY5Yneurons transfected with targeted ChABC had significantly longer neurites than control (non-transfected) neurons, mean length 274.5μm compared to 68.9μm for controls, Z = 8.92, P<0.0001 and neurons transfected with non-targeted ChABC, mean neurite length 119.6μm, Z = 6.54, P = 0.0001. Neurons transfected with non-targeted ChABC had significantly longer neurites than controls Z = 8.63, P = 0.0001. B) The number of branches extending from a single neuron were also higher in cells transfected with targeted ChABC, compared to controls, z = 4.38, P<0.0001 and neurons transfected with non-targeted ChABC, z = 2.47, P = 0.014. There were no differences in the number of neurites/cell between cells expressing non-targeted ChABC and controls z = 0.04, P = 0.96.Values shown are mean +/-SEM. C) Neurons stained with β-tubulin, sprouting in cultures of neurons transfected with targeted ChABC are indicated by circles. All comparisons by MWU-test with a Holm adjustment, ***P<0.001, **P<0.01, n = 36. Each experiment was repeated three times.

Of particular note, are the remarkably long neurites seen on some neurons expressing targeted ChABC, which are almost 20 times the length of those of the GFP-transfected cells (S2 Fig). These constitute ~10% of the neurons present, and have only been observed in cell cultures transfected with the axon-targeted construct. Furthermore, comparison of neurite lengths of cultures transfected with targeted with those transfected with non-targeted ChABC, showed that cultures expressing targeted ChABC had significantly longer neurites, supporting the view that targeting ChABC to the axon markedly enhances its ability to promote axon outgrowth (Fig 1).

In this study, there was no significant difference in the number of neurites per cell between controls and neurons transfected with the non-targeted construct. This contrasts with the results observed *in* vivo [5] and may be due to a lower transfection efficiency obtained with plasmid transfection used here, compared to that obtained via lentiviral transduction, used in the *in vivo* study.

We also conducted an additional experiment to determine if the results obtained with our neuronal cell line, are recapitulated in cultures of primary neurons. To this end we analysed the effect of targeted ChABC on neurite outgrowth of dissociated dorsal root ganglion cells plated onto CSA. S3 Fig shows that targeted ChABC also enhances neurite outgrowth of these primary neurons compared to both controls and to non-targeted ChABC.

### SH-SY5Y cells produce CSPGs

**Fig 2:**
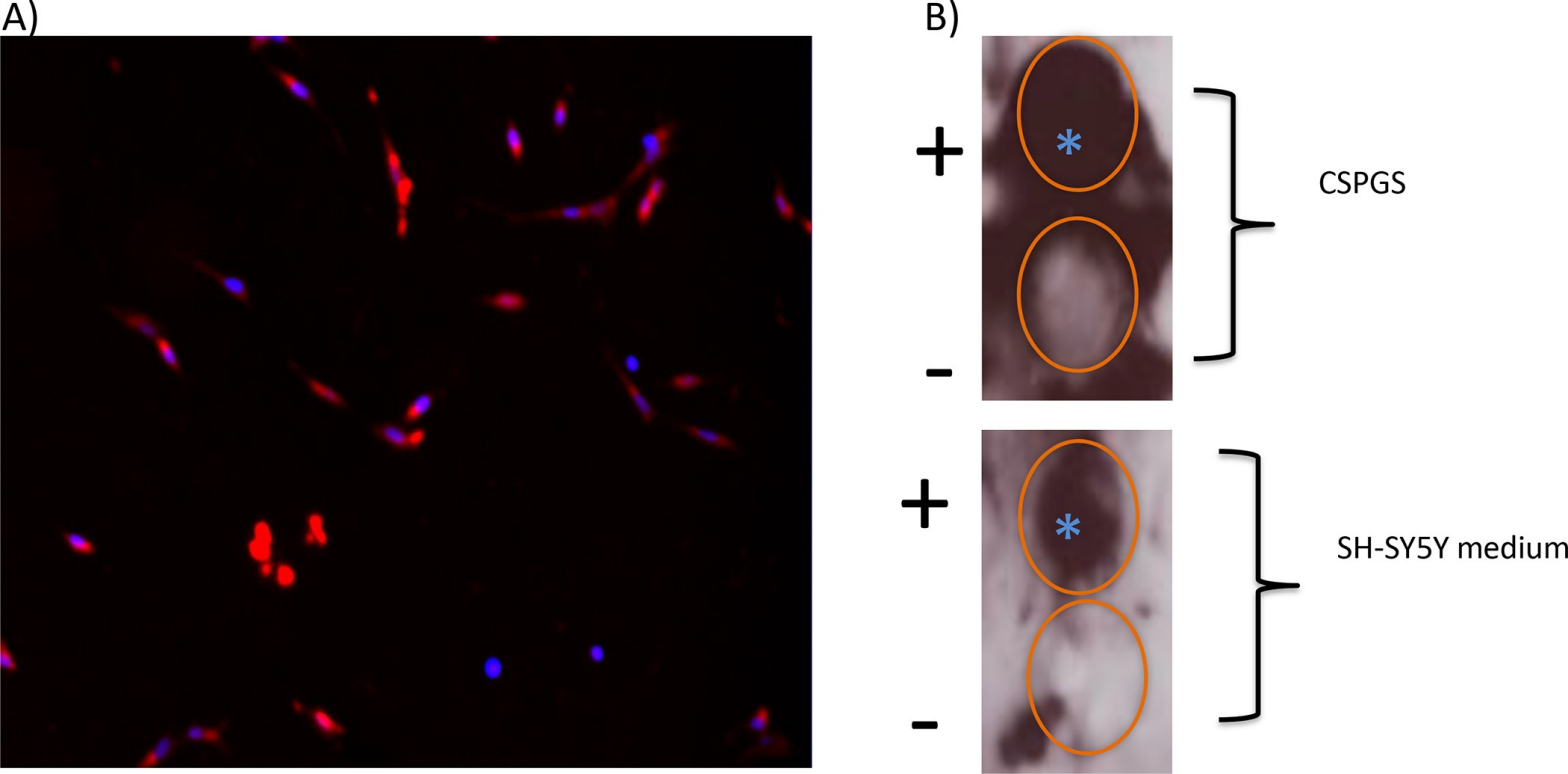
Detection of endogenously produced CSPGs by SH-SY5Y cells. A) CS56-staining (red) showing that CSPGs are present on the surface of these neurons. B) Dot blot of control CSPGs without (-) and with (+) ChABC-digestion, (top two panels) and conditioned medium from SH-SY5Y neurons without (-) and with (+) ChABC–digestion. The blot was stained with antibody 2B6, which detects an epitope exposed after ChABC-digestion. (+) indicates that ChABC was added 3h prior to antibody staining. (-) indicates undigested with ChABC. The blue star indicates positive staining.

We extended our studies to investigate the mechanism underlying the enhancement of neurite outgrowth by axon-targeted ChABC on laminin. We show that SH-SY5Y cells produce and shed CSPGs. Fig 2A shows these neurons stain with an antibody to CS56, which recognizes intact CSPGs. Since these cells were not permeabilised prior to staining, this is consistent with the presence of intact CSPGs on their surface. Fig 2B is a dot blot of medium from SH-SY5Y cells, probed with an antibody, 2B6. This recognizes an epitope exposed following digestion of CSPGs with ChABC. It can be seen that medium from the cells and that of the positive control (CSPGs purchased from Millipore) stain with the antibody following enzyme digestion, confirming the presence of CSPGs in the medium.

The Blyscan assay is a sensitive quantitative dye binding method for detecting sulphated glycosaminoglycans and a measure of total sulphated glycosaminoglycan content. The dye, 1,9-dimethlmethylene blue is a specific label for the sulphated polysaccharide component of proteoglycans. Using this assay we show that CSPGs were also present in the conditioned medium of SH-SY5Y cells at ~4.0μg/ml. These results confirm that SH-SY5Y neurons produce CSPGs, and are consistent with the hypothesis that ChABC-axon enhances neurite outgrowth on laminin by digesting the CSPGs produced by these neurons. The magnitude of the effect on axon length suggest that endogenously produced CSPGs provide a significant block to neurite outgrowth.

### ChABC expression in neurons up-regulates cell surface expression of β-integrin

**Fig 3:**
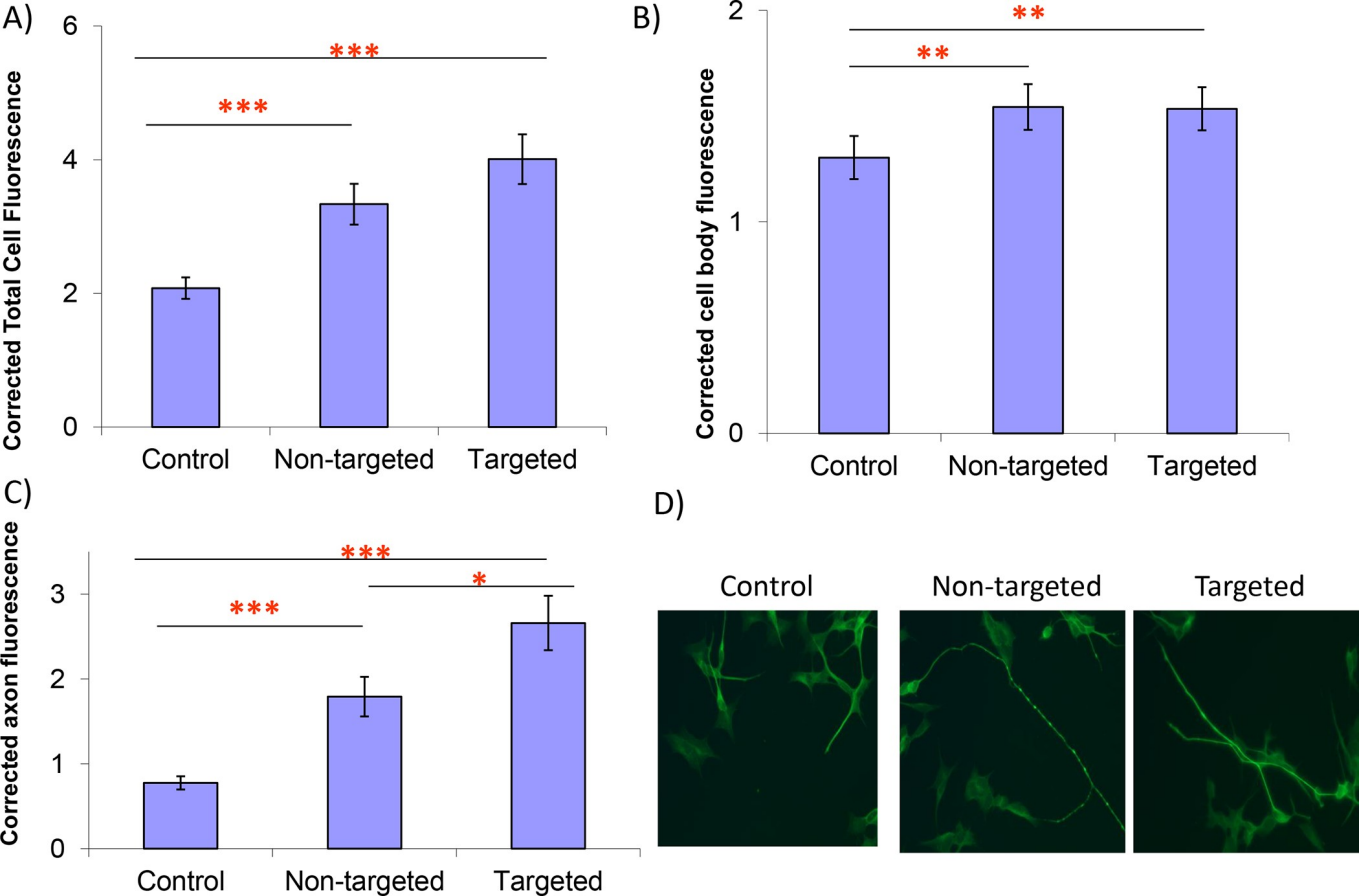
Surface β-integrin expression of neurons plated onto CSA: SH-SY5Y neurons transfected with the different ChABC constructs and non-transfected controls. A) Average total cell fluorescence for β-integrin. Fig 3A shows that total cell fluorescence is significantly increased in neurons expressing targeted ChABC compared to the controls, mean pixel intensity 4.01 compared to 2.1 for controls, Z = 4.91, P = 0.0001, and also that of non-targeted ChABC, mean pixel intensity 3.33 was higher than that of the controls, Z = 3.51, P = 0.0001, although there were no differences between the targeted and non-targeted constructs Z = 1.38, P = 0.2. B) Average cell body fluorescence. Fig 3B shows that cell body fluorescence was higher in neurons transfected with both targeted and non-targeted ChABC compared to controls, mean pixel intensity 1.54 and 1.53 respectively, compared to 1.3 for controls, Z = 2.95, P = 0.003, z = 3.07, P = 0.002. Again there was no difference in pixel intensity between neurons expressing targeted or non-targeted ChABC, Z = 0.12, P = 0.9. C) Average axon fluorescence. Fig 3C shows that β-integrin fluorescence in the axonal compartment is significantly greater in cells transfected with targeted ChABC, compared to controls, mean pixel intensity 2.66 compared to 0.77, Z = 6.01, P = 0.0001. It is also higher in cells expressing non-targeted ChABC compared to controls, mean pixel intensity 2.66, Z = 4.11, P = 0.0001.Additionally, axonal fluorescence was higher in neurons transfected with targeted ChABC compared to neurons expressing the non-targeted version, z = 2.23, P = 0.03. D) β-integrin-stained SH-SY5Y neurons, showing the presence of long neurites in the ChABC transfected cultures. Values shown are mean +/-SEM, MWU-test.*P<0.05, **P<0.01, ***P<0.001, n = 36/group. Each experiment was repeated three times.

Further investigation of the mechanisms responsible for the enhancement of neurite outgrowth showed that the cell adhesion molecule β1-integrin, is up-regulated at the surface of neurons into which the ChABC gene is introduced. Fig 3 shows that total cell, cell body and axonal integrin expression are significantly increased in neurons expressing targeted or non-targeted ChABC compared to the controls. Importantly, we show that β-integrin expression is significantly higher in the axonal compartment of neurons transfected with targeted ChABC compared to non-targeted ChABC. Thus ChABC expression in the axon, enhances expression of β1-integrin at a location where it is optimally sited to promote neurite outgrowth. This is consistent with the targeted form of ChABC being more effective at promoting axon out-growth than the non-targeted version. Indeed, the very long neurites seen in cultures transfected with targeted ChABC show strong staining for β-integrin (S4 Fig).

To determine whether the enhancement of neurite outgrowth observed in primary cultures of DRGs transfected with targeted ChABC was also associated with integrin-mediated mechanism we measured integrin levels in transfected DRG neurons. The antibody used to stain the SH-SY5Y cells functioned poorly with the DRGs, so integrin expression was measured indirectly by staining for phosphorilated focal adhesion kinase (pFAK).

Integrins can exist in both activated and inactivated forms. When integrins are activated, focal adhesion kinase (FAK) becomes phosphorylated (pFAK). Therefore, importantly, we have demonstrated the presence of pFAK in primary cultures of neurons transfected with the targeted version of ChABC, S5 Fig. This is consistent with the β-integrin molecules being in an activated state, and thus competent to promote neurite outgrowth, via an increase in cell adhesion.

### RhoA is diminished by ChABC expression

**Fig 4:**
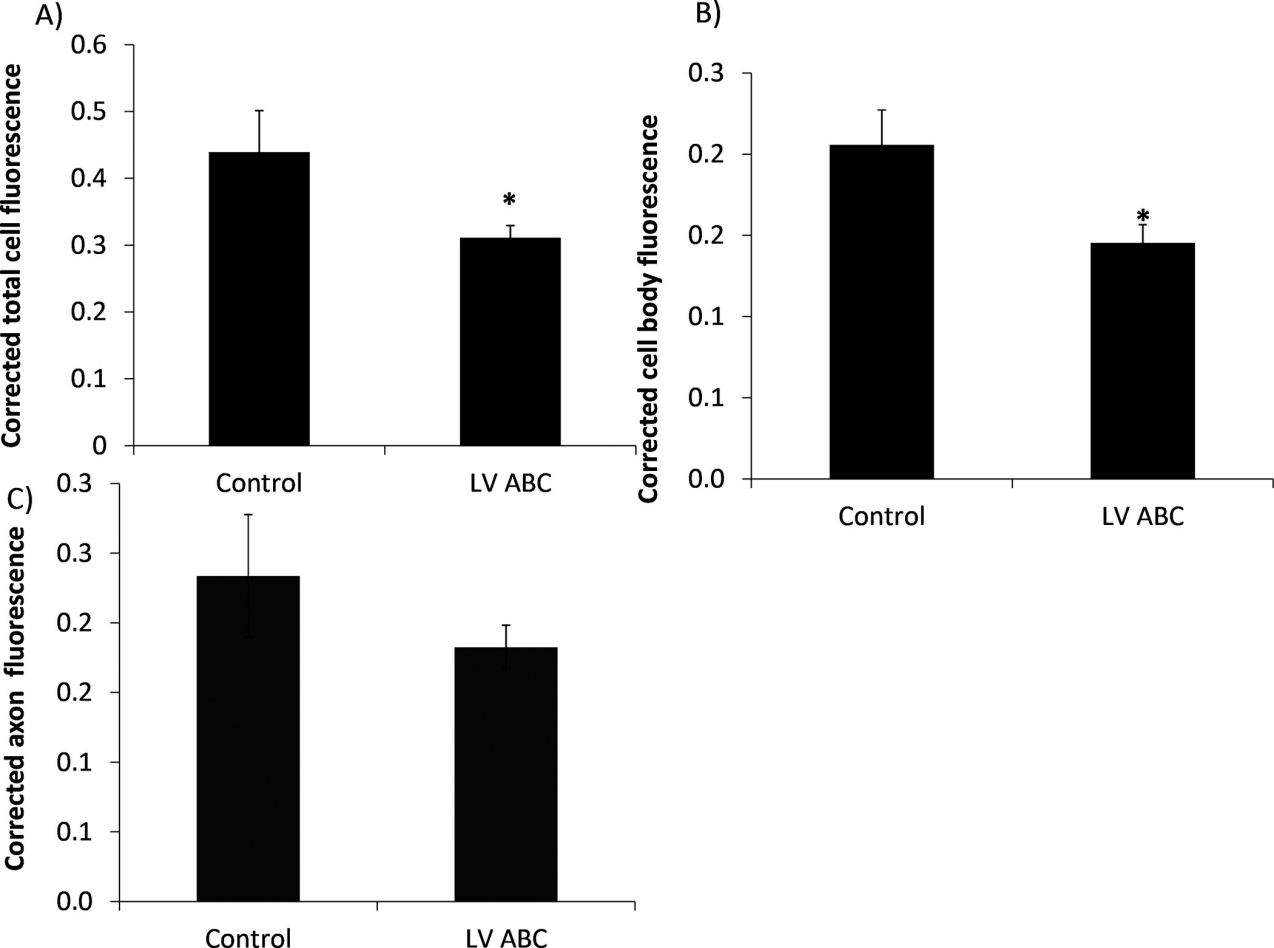
RhoA staining of SH-SY5Y neurons transfected with non-targeted ChABC or GFP and plated onto CSA. Total RhoA protein levels are decreased in neurons, mean pixel intensity 0.44 compared to 0.31, (panel A), Z = -2.0, P = 0.046 and the cell bodies, mean pixel intensity 0.21 compared to 0.15 (panel B), z = -2.44, p = 0.015 of neurons expressing ChABC, compared to GFP transfected controls. There was no difference in axonal RhoA fluorescence, mean pixel intensity 0.18 compared to 0.23, z = -1.07, P = 0.29, (panel C). Values are mean +/-SEM, MWU-test. *P<0.05, 36/samples/group. Each experiment was repeated 3 times.

RhoA is a potent inhibitor of axon regeneration [26]. Interestingly, we found that ChABC expression altered the intracellular levels of RhoA. Fig 4 shows that RhoA staining is reduced in neurons expressing ChABC.

Moreover, when ChABC expression is directed to the axon, RhoA staining is additionally reduced in the axonal compartment, S6 Fig.

This suggests that ChABC has to be targeted to the axon in order to decrease RhoA expression in this compartment. These findings identify an additional mechanism for ChABC-mediated promotion of axon outgrowth, and again expression of the enzyme in the axonal compartment would be predicted to give optimal performance.

### SH-SY5Y neurons expressing ChABC show a reduction the intracellular levels of PTEN

**Fig 5:**
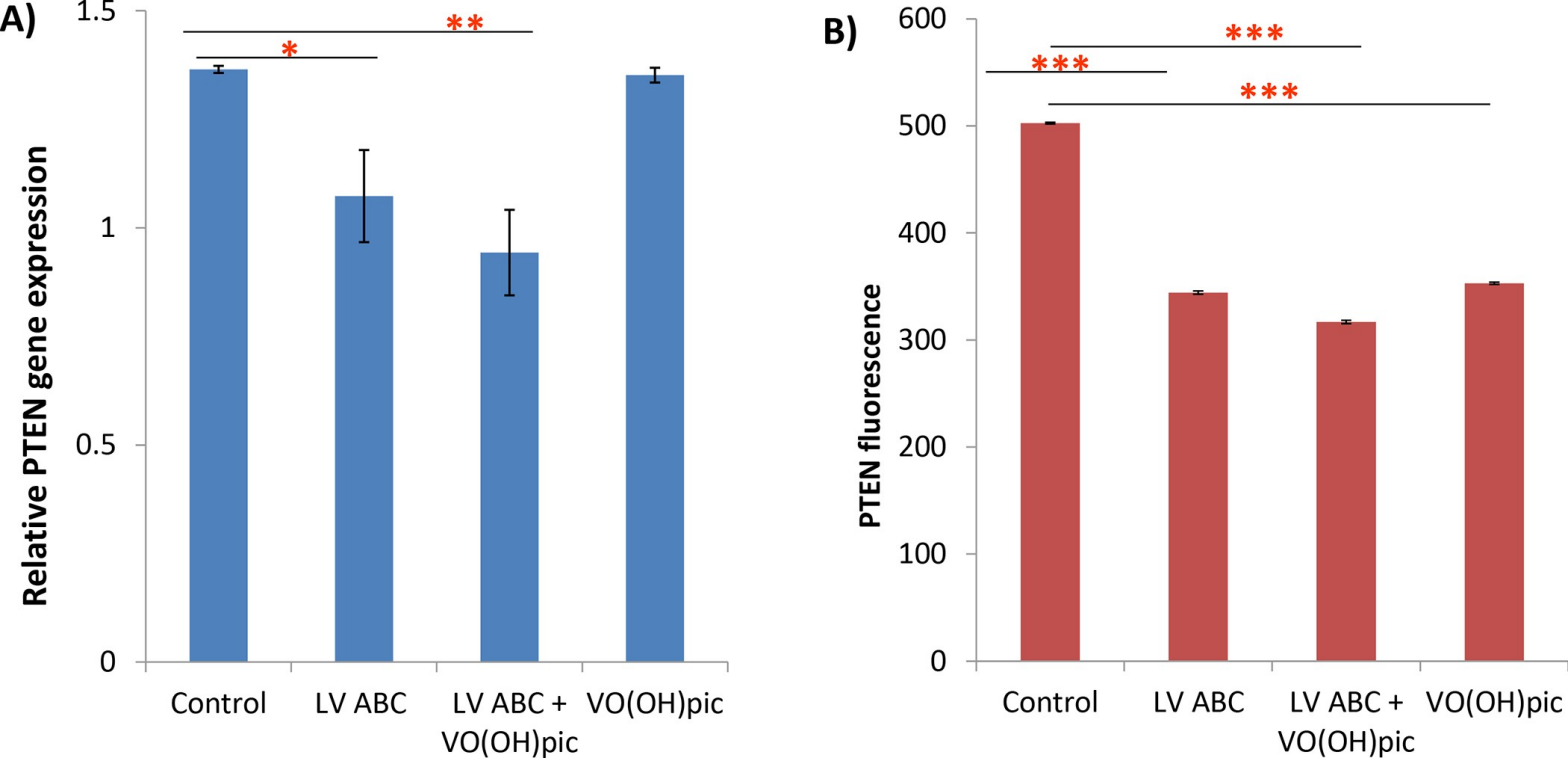
PTEN gene and protein expression in ChABC-transduced SH-SY5Y neurons and controls. A) PTEN gene expression is reduced in neurons expressing ChABC alone, mean value 1.07, compared to controls mean value,1.37, Z = -2.17, P = 0.030, and neurons expressing ChABC in the presence of the PTEN inhibitor VO(OH)pic, mean value 0.94, Z = -2.59, P = 0.008, but unaffected by the PTEN inhibitor VO(OH)PIC (alone), mean value 1.35, Z = -0.27, P = 0.837, n = 36 B) The reduction in PTEN gene expression, seen in the ChABC transduced cells, is accompanied by a reduction in the intracellular levels of PTEN protein, ChABC alone mean pixel intensity 344.22 compared to 502.65 in controls, Z = -17.30, P<0.0001, ChABC +VO(OH)pic, mean pixel intensity 316.8, Z = -17.30, P<0.0001. The PTEN inhibitor alone also, unexpectedly, resulted in a drop in PTEN protein levels compared to controls, mean pixel intensity 353.01, Z = -17.30, P<0.0001, n = 200. Values shown are mean +/-SEM, MWU-Test with Holm adjustment. *P< 0.05, **P<0.01, ***P<0.001. Each experiment was repeated 3 times.

SH-SY5Y cells were transduced with a lentivirus encoding non-targeted ChABC [5], or treated with the PTEN inhibitor, VO (OH)Pic [25]. A third group was transduced with the lentivirus encoding ChABC and treated with the PTEN inhibitor. PTEN gene expression was significantly reduced in cells expressing ChABC (non-targeted), Fig 5A, and this was accompanied by a decrease in PTEN protein expression, Fig 5B.

### A reduction in intracellular levels of PTEN, is accompanied by an increase in neurite outgrowth

The increase neurite length observed in ChABC expressing neurons is similar to that produced by the PTEN inhibitor VO(OH)PIC [25]. Moreover, a combination of ChABC and the PTEN inhibitor did not produce a further increase neurite length, suggesting that CSPGs, like myelin inhibitors, block neurite outgrowth via a pathway involving PTEN (Table 2).

**Table 2:** The effect of ChABC (non-targeted) and the PTEN inhibitor VO(OH)Pic, on neurite lengths.

Group	Neurite length μm	95%CI	P value (compared to control)
Control	62.80	59.02–66.82	
LVABC	68.72	64.33–73.41	0.022
LV ABC + VO(OH)Pic	70.11	65.36–75.19	0.045
VO(OH)Pic	68.72	64.07–73.70	0.049

SH-SY5Y neurons transduced with a lentivirus encoding ChABC, and those treated with the PTEN inhibitor, have longer neurite lengths than controls. A combination of ChABC and the PTEN inhibitor did not enhance neurite length over ChABC alone. Data are the mean (μm) and confidence intervals. n = 6/group. The experiment was repeated 3 times.

## Discussion

Addition of the peptide containing the YENPTY motif targets proteins with the tag to the axonal compartment of a neuron [27]. This was added to the modified ChABC gene construct [14], along with a tetracystine tag to allow identification of successfully transfected cells by FIAsH staining. This tag worked well in DRG neurons, but unexpectedly, gave high background staining in the SH-SY5Y cells. Therefore, the results from this study, represented measurements from all neurites. An estimated transfection efficiency for this cell line, using ChABCmcherry [28], which is a similar size to the construct used, was ~10%. SH-SY5Y neurons, transfected with targeted ChABC, exhibited greatly enhanced neurite lengths, compared to controls, (both GFP-transfected and non-transfected). Interestingly, this was noted both on CSA and on laminin substrates. Some of the neurites (~10%), were remarkably long; one measured 194um, almost 20 times the average neurite length of control neurons recorded during this set of experiments. The number of neurites/cell was also increased in these cultures, suggesting that the cells’ intrinsic ability to generate neurites was enhanced. Transfection of SH-SY5Y neurons with non-targeted ChABC, also resulted in an enhancement of neurite length, as expected, but not in sprouting. The latter finding (no enhanced sprouting) is in contrast to what has been observed *in* vivo [5] but could be explained by the much higher transduction efficiencies obtained with lentiviruses (usually ~80–90%), compared to the plasmid transfection efficiencies used in this study, which were estimated to be ~10%. Another possibility is that the enhanced sprouting observed *in vivo*, is a result of ChABC-mediated dissolution of perineuronal nets, structures not present in our SH-SY5Y cultures. The observation that targeting ChABC to the axon enhances the sprouting potential of ChABC is an important finding, as the ability to promote plasticity is a key mechanism of promoting repair following SCI [6]. Using the same culture system (SH-SY5Y neurons), we have previously shown that targeting ChABC to the neuronal growth cone, also potentiates its ability to promote neurite outgrowth compared to non-targeted ChABC [28]. However, unexpectedly, the magnitude of the effect is much greater when ChABC is targeted to the axonal compartment of neurons. Whilst growth cone-targeted ChABC doubled the average neurite length on CSA compared to non-targeted ChABC [28], targeting ChABC to the axon increased the average neurite length by a factor of ~3.7. This difference is likely due to its distribution all along the axon, as opposed to the much smaller area of the growth cone.

We show that SH-SY5Y neurons produce CSPGs which are shed into the medium. Therefore, it is likely that the increase in neurite length of the cultures transfected with targeted ChABC, compared to the GFP transfected cells, seen on a laminin substrate, which is normally permissive to neurite outgrowth, is due in part to ChABC digestion of neuron-derived CSPGs. The difference in neurite length between GFP-transfected cells and those transfected with targeted ChABC plated on laminin is significant, suggesting that neuron-derived CSPGs may provide an additional block to neurite outgrowth. This finding has direct relevance to the regeneration of cortical neurons, as they also produce CSPGs, which are located on the cell surface and shed into the extracellular matrix [29]. This provides a mechanism by which ChABC could specifically promote regeneration of the corticospinal tract. Targeted ChABC also enhances axon outgrowth of DRGs plated on CSA, compared to both GFP controls and non-targeted ChABC, demonstrating the effect is recapitulated in primary neurons. However, the magnitude of the effect is smaller than that observed with the SH-SY5Y neurons.

We show that ChABC upregulates β-integrin at the cell surface of SH-SY5Y neurons. Moreover, β-integrin expression is further up-regulated on axons when neurons are transfected with the targeted form of the enzyme. This is consistent with the targeted form of ChABC being more effective at promoting axon outgrowth than the non-targeted version.

β-integrins can exist in both activated and inactivated forms. Therefore, importantly, we have demonstrated that the enhanced staining for β1-integrin is accompanied by an increase in staining for pFAK, consistent with the β-integrins being in the activated state. They would therefore be able to promote neurite outgrowth via an increase in cell adhesion. CSPGs are known to down-regulate cell surface integrin expression [30] and also to inactivate integrins [31] which are required for regeneration [32]. This provides a likely mechanism for the ChABC-mediated effect on neurite outgrowth. Additionally, CSPGs promote the translation of RhoA in axons [33]. RhoA is a potent inhibitor of axon regeneration [26], thus, this may be another key mechanism by which CSPGs block neurite outgrowth. RhoA staining is reduced in neurons expressing ChABC (non-targeted) and this becomes detectable in the axonal compartment, when neurons (DRGs) are transfected with the construct where ChABC is targeted to the axon. This provides a further mechanism for ChABC-mediated promotion of axon outgrowth. Again, expression of the enzyme in the axonal compartment would be predicted to give optimal performance, consistent with the enhanced performance of the targeted version of ChABC in promoting neurite outgrowth. It is of interest, also to note, that targeting a soluble form of adenyl cyclase to the axonal compartment of rat DRGs, promotes neurite outgrowth on CSPGs [34]. This raises the possibility, that targeting growth promoting molecules to the axon, rather than the cell as a whole, may be a more effective approach for promoting regeneration.

We have demonstrated a role for PTEN in ChABC mediated neurite outgrowth.

PTEN mRNA and protein expression were significantly reduced in cells expressing ChABC, and this was accompanied by the increase neurite length. A similar increase in neurite length was produced by the PTEN inhibitor VO-OHpic [25]. This is a vanadium-based potent inhibitor of PTEN which, unlike some of the other vanadium based inhibitors, is highly specific for PTEN [35]. VO-OHpic had no effect on PTEN mRNA levels, suggesting that blocking PTEN function doesn’t result in any feedback loop to PTEN transcription. It did however, cause a significant drop in PTEN protein levels. This suggests that in addition to blocking PTENs lipid phosphatase function, it also causes destabilization of the PTEN protein. A combination of ChABC and the PTEN inhibitor did not increase neurite length further. This implies that ChABC promotes neurite outgrowth on CSA via a PTEN-dependent mechanism and that CSPGs, in common with myelin inhibitors [36], block neurite outgrowth, via a pathway involving PTEN. This is a significant finding, as the there is accumulating evidence that the PTEN pathway is critical for the regeneration of adult cortical neurons [37,38, 39]. Deletion of PTEN has been additionally been reported to increase sprouting of adult corticospinal neurons [38]. Therefore, ChABC may promote sprouting via its effect on neuronal PTEN levels.

Although the enhancement of neurite outgrowth associated with PTEN inhibition is modest, it is the first report of ChABC enhancing neurite outgrowth via an intrinsic mechanism. This finding, taken together with the upregulation of cell-surface β1-integrin, adds to the list of the enzymes’ known benefits and strengthens the case for including ChABC as an essential component of any combination treatment for SCI.

The observation that targeting ChABC to the axon, enhanced neurite length compared to non-targeted ChABC, suggests that this may be an even more powerful way of enhancing axon regeneration. Importantly, the finding that ChABC down-regulates PTEN in neurons, and promotes neurite outgrowth of neurons expressing surface CSPGs on laminin, has important implications for promoting long-distance regeneration of the corticospinal tract. This is still a major challenge that remains to be overcome, before a successful treatment for SCI is attained. Indeed, the combination of PTEN knockdown, with CSPG removal has been predicted to be a promising strategy to promote extensive plasticity in adult mammals [36]. In summary, the encouraging findings reported here, suggest that this novel axon-targeted variant of ChABC holds great promise as a treatment for SCI and warrants further investigation in an *in vivo* study to reveal its true potential.

## Supporting information

S1 Table Primers used for qPCR. (DOCX)

S1 Fig Effect of different concentrations of CSA on neurite outgrowth by SH-SY5Y neurons. Neurons were stained with anti-β-tubulin-III to visualise the neurites. The MWU-test showed a significantly lower median neurite length on a substrate containing laminin+ CSA 75μg/ml compared to laminin alone, P<0.001, n = 230, indicating that this concentration of CSA is inhibitory to neurite outgrowth. L = laminin, C4S = chondroitin-4-sulfate (CSA).
(TIF)

S2 Fig Neurons transfected with axon-targeted chABC and stained for β-III tubulin. The neurite indicated by the arrow is ~ 194μm. The colour represents pixel intensity, as shown in the scale to the left of the figure, where white is maximum intensity and black is minimum.
(TIF)

S3 Fig Neurite outgrowth by dissociated DRGs transfected with GFP or the different chABC constructs and plated onto CSA. The Neurites of DRG’s transfected with targeted ChABC and plated onto CSA, mean neurite length, 283.6μm, are significantly longer than those of DRGs transfected with non-targeted ChABC, mean length 255.0μm, plated on CSA, Z = 16.13, P<0.0001, and the GFP controls, mean length 209.78μm, plated on CSA, z = 17.3, P<0.0001. The neurites of DRGs transfected with non-targeted ChABC and plated onto CSA are also significantly longer than the GFP controls, z = 17.3, P<0.0001. Values are mean+/-SEM. MWU test with Holm adjustment. n = 200.
(TIF)

S4 Fig An example of the long neurites observed in cultures of neurons (SH-SY5Y cells) transfected with axon-targeted chABC, plated onto CSA and stained for β-integrin. Long neurites are associated with enhanced staining for β-integrin.
(TIF)

S5 Fig **DRGs plated onto CSA and transfected with targeted chABC (Bottom panels, B) or GFP (Top panels, A) and stained for β-tubulin (Left hand panels) or pFAK (Right hand panels).** Left hand panel (Top) shows staining for β-tubulin III is present in both the cell body and the axons of controls, (A) and targeted ChABC transfected DRGs (bottom panel), B. Right hand panel (Top), A shows weak staining for pFAK in the cell body of GFP-transfected DRGs and no staining is observed in the axonal compartment. In contrast, targeted ChABC transfected DRGs show bright staining for pFAK in the cell bodies and diffuse staining in the axonal compartment, indicating β-integrin activation, (bottom right hand panel, B).
(TIF)

S6 Fig **Rho A-staining of DRGs plated onto CSA and transfected with targeted chABC (bottom panel) or GFP (Top panel).** Left hand panels show staining for β-tubulin III is present in both the cell body and the axons of controls (Top) and DRGs transfected with targeted ChABC(bottom). Right hand panels show strong staining for RhoA in both the cell body and axons of control neurons (Top). Neurons transfected with targeted ChABC show staining for RhoA in the cell body, but weak staining of the axonal compartment (bottom).
(TIF)

S1 File Raw data. (ZIP)
