# Supplementary material for: Targeting chondroitinase ABC to axons enhances the ability of chondroitinase to promote neurite outgrowth and sprouting
Source: PLoS One. 2020 Jan 21;15(1):e0221851. doi: 10.1371/journal.pone.0221851 (PMC6974052; doi:10.1371/journal.pone.0221851)
Supplement: S1 Table — (DOCX) [file pone.0221851.s001.docx]

| Gene | Reverse primer | Forward primer | Roche probe |
| --- | --- | --- | --- |
| PTEN (NM_000314.4) | 3’gcacagaggccctagatttc’5 | 3’cgcctctgactgggaatagt’5 | 60 |
| ACTB (NM_001101.3) | 3’ ccaaccgcgagaagatga’5 | 3’ ccagaggcgtacagggatag’5 | 64 |

Abbreviations: PTEN=Phosphatase and tensin homologue deleted on chromosome10. ACTB=β-actin. All primers were intron spanning.
