# Supplementary figures and images for: Targeting chondroitinase ABC to axons enhances the ability of chondroitinase to promote neurite outgrowth and sprouting
Source: PLoS One. 2020 Jan 21;15(1):e0221851. doi: 10.1371/journal.pone.0221851 (PMC6974052; doi:10.1371/journal.pone.0221851)

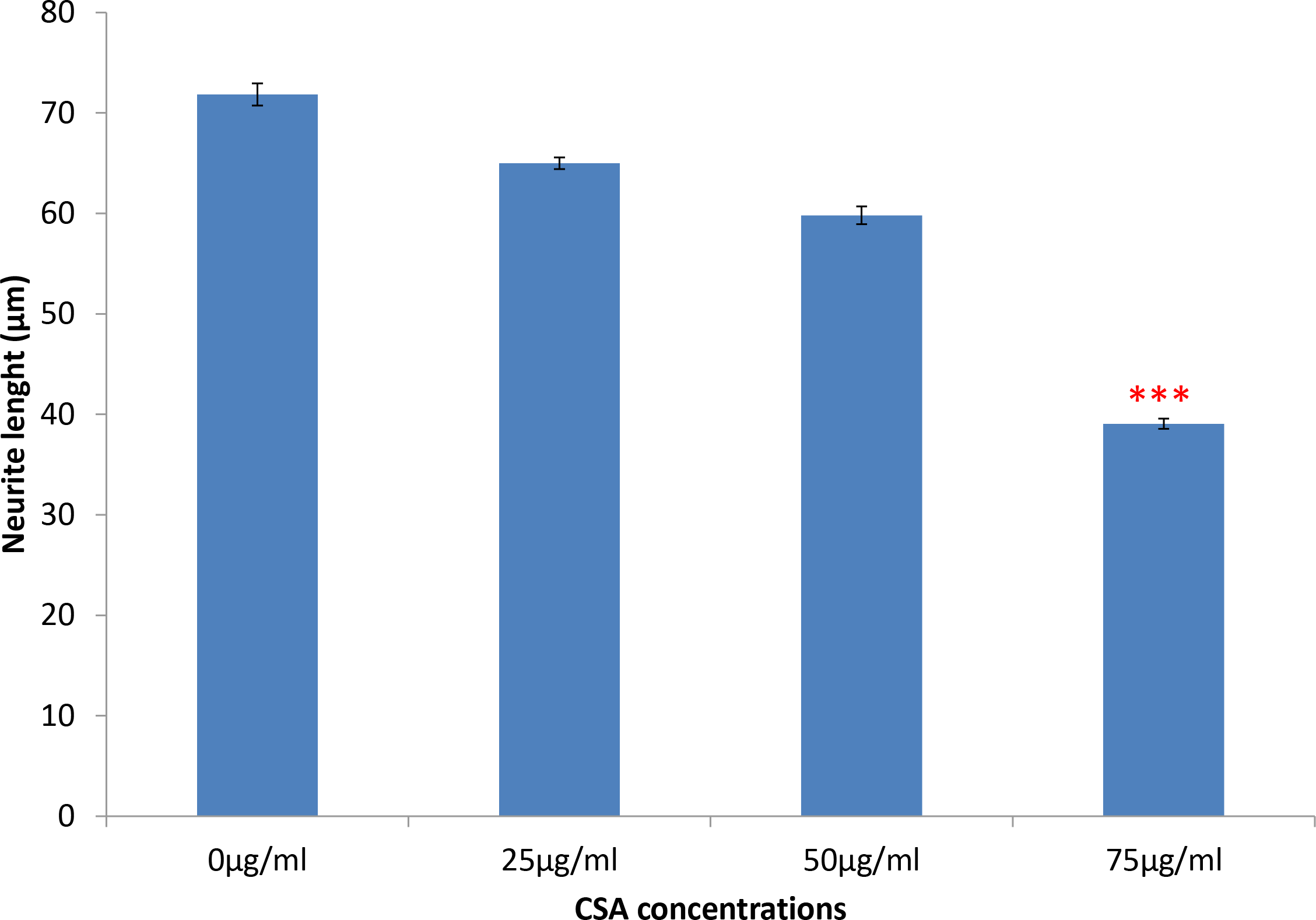

Supplement: S1 Fig — Neurons were stained with anti-β-tubulin-III to visualise the neurites. The MWU-test showed a significantly lower median neurite length on a substrate containing laminin+ CSA 75μg/ml compared to laminin alone, P<0.001, n = 230, indicating that this concentration of CSA is inhibitory to neurite outgrowth. L = laminin, C4S = chondroitin-4-sulfate (CSA). (TIF) [file pone.0221851.s002.tif]

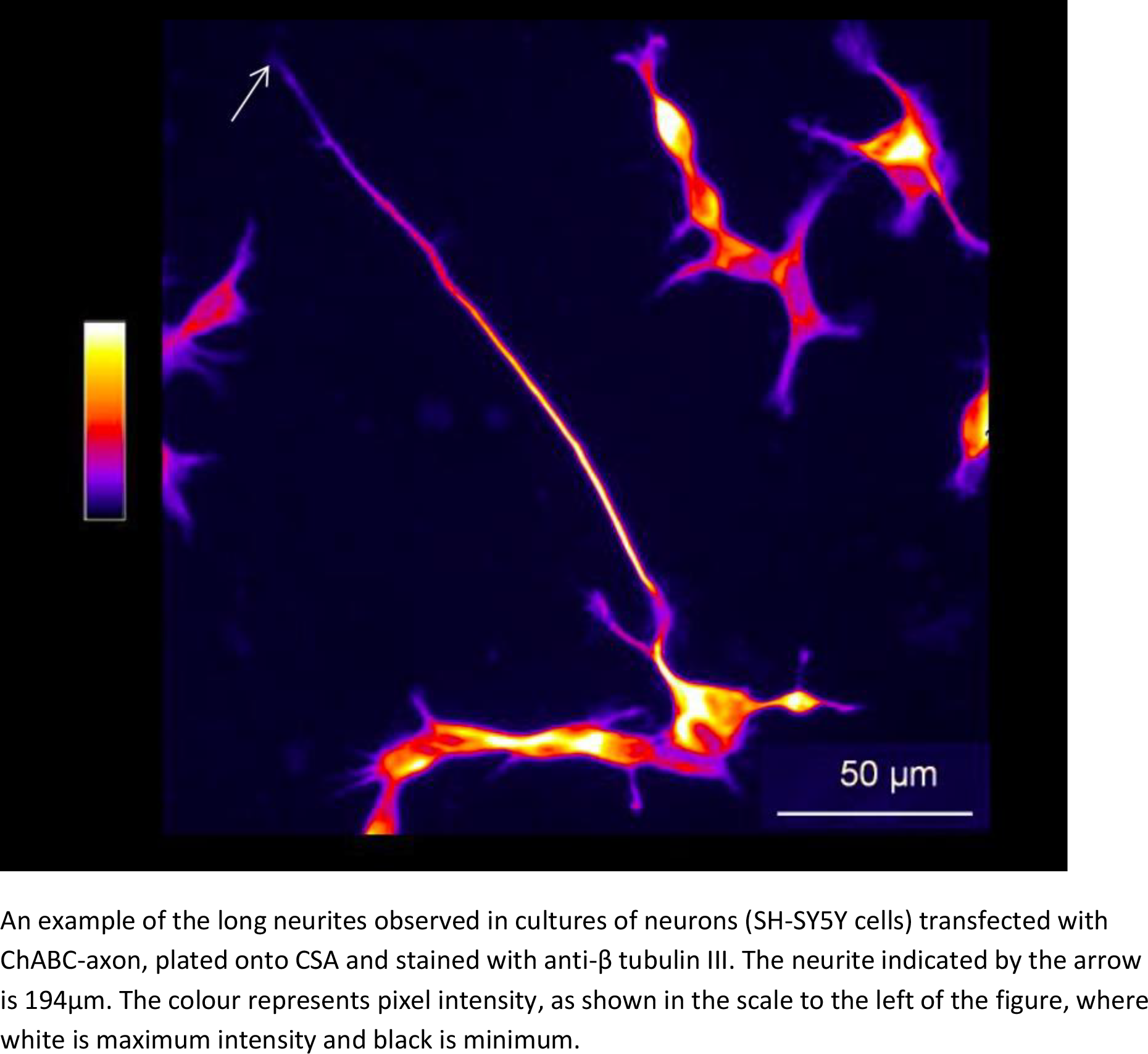

Supplement: S2 Fig — The neurite indicated by the arrow is ~ 194μm. The colour represents pixel intensity, as shown in the scale to the left of the figure, where white is maximum intensity and black is minimum. (TIF) [file pone.0221851.s003.tif]

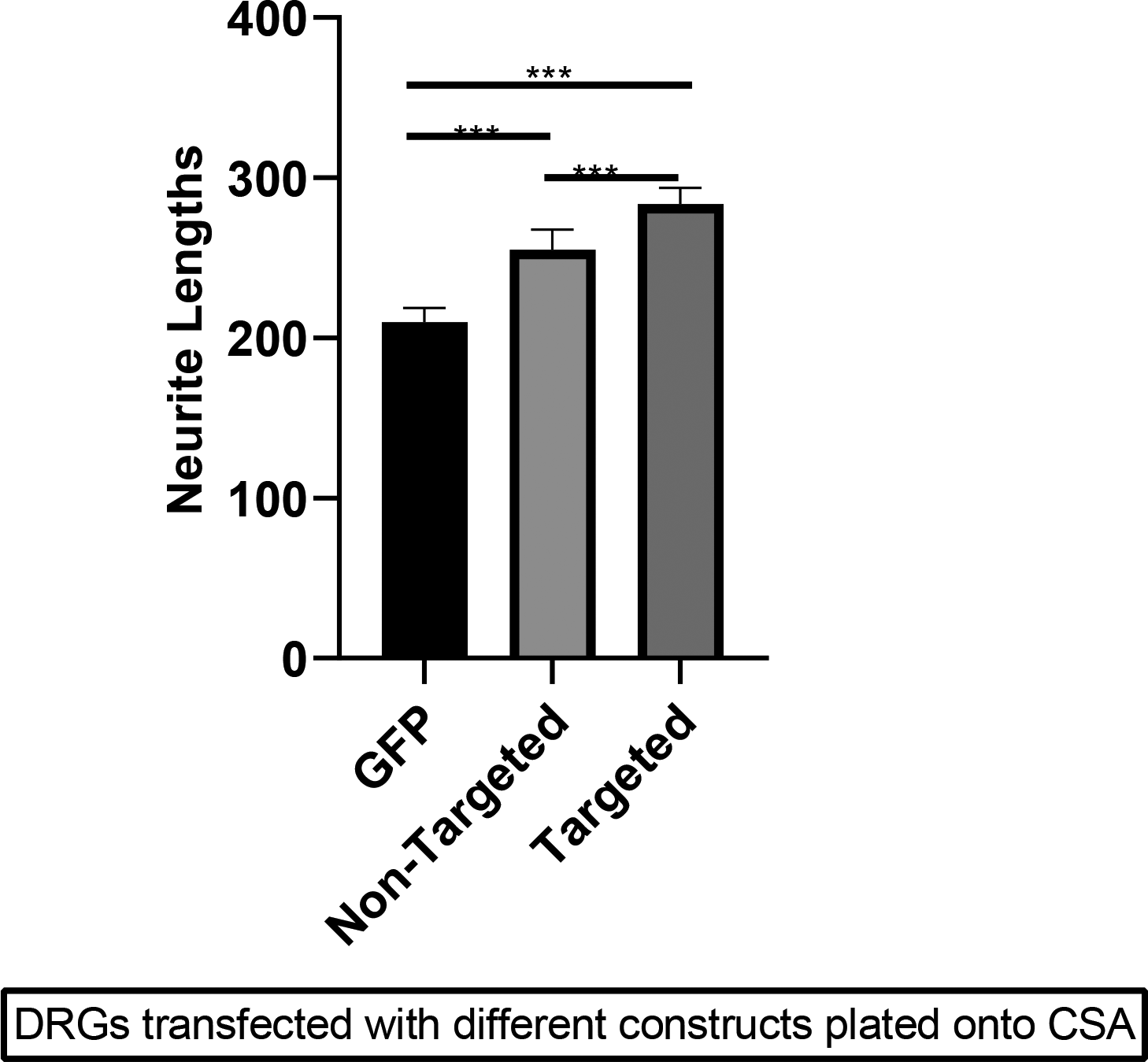

Supplement: S3 Fig — The Neurites of DRG’s transfected with targeted ChABC and plated onto CSA, mean neurite length, 283.6μm, are significantly longer than those of DRGs transfected with non-targeted ChABC, mean length 255.0μm, plated on CSA, Z = 16.13, P<0.0001, and the GFP controls, mean length 209.78μm, plated on CSA, z = 17.3, P<0.0001. The neurites of DRGs transfected with non-targeted ChABC and plated onto CSA are also significantly longer than the GFP controls, z = 17.3, P<0.0001. Values are mean+/-SEM. MWU test with Holm adjustment. n = 200. (TIF) [file pone.0221851.s004.tif]

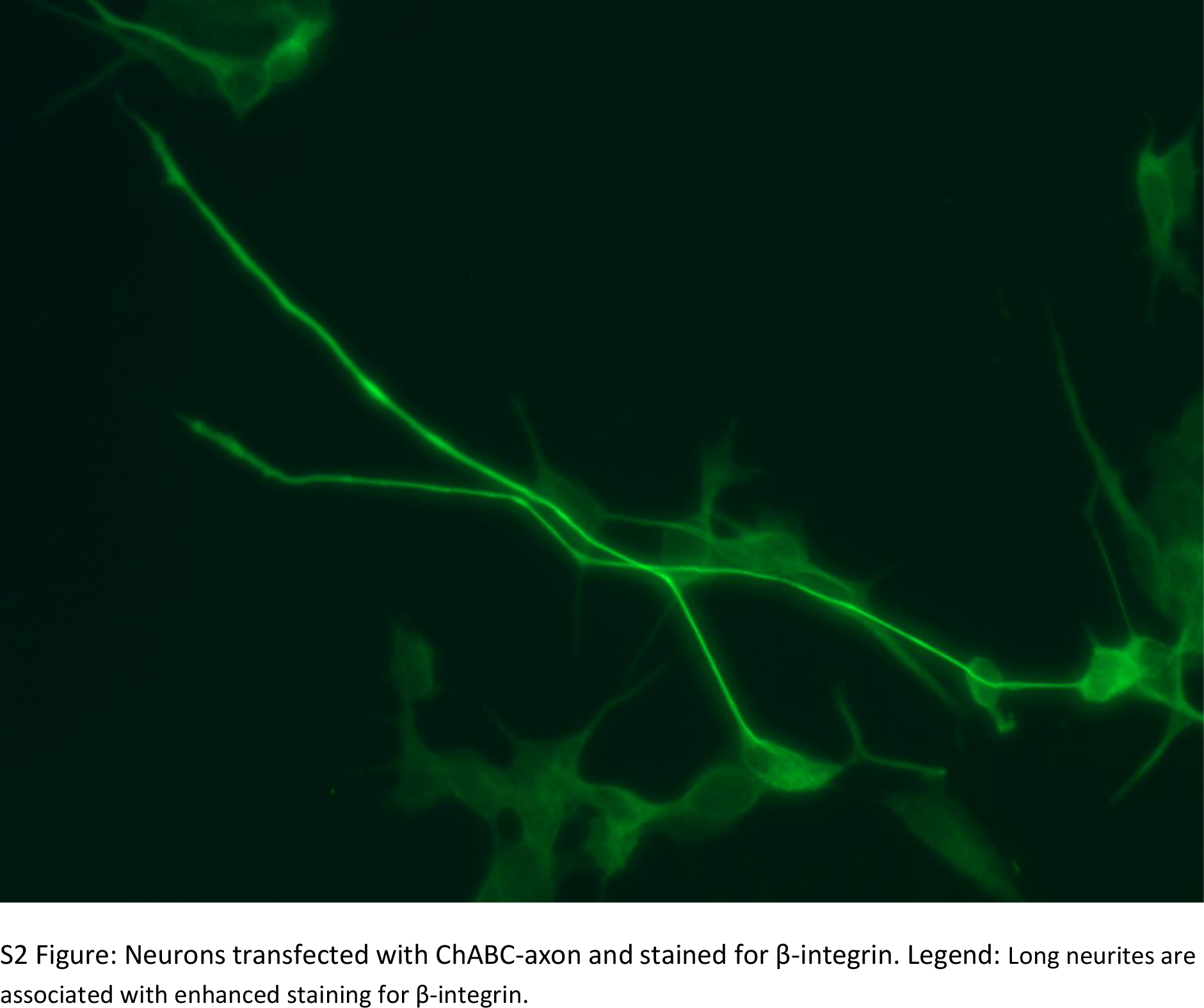

Supplement: S4 Fig — Long neurites are associated with enhanced staining for β-integrin. (TIF) [file pone.0221851.s005.tif]

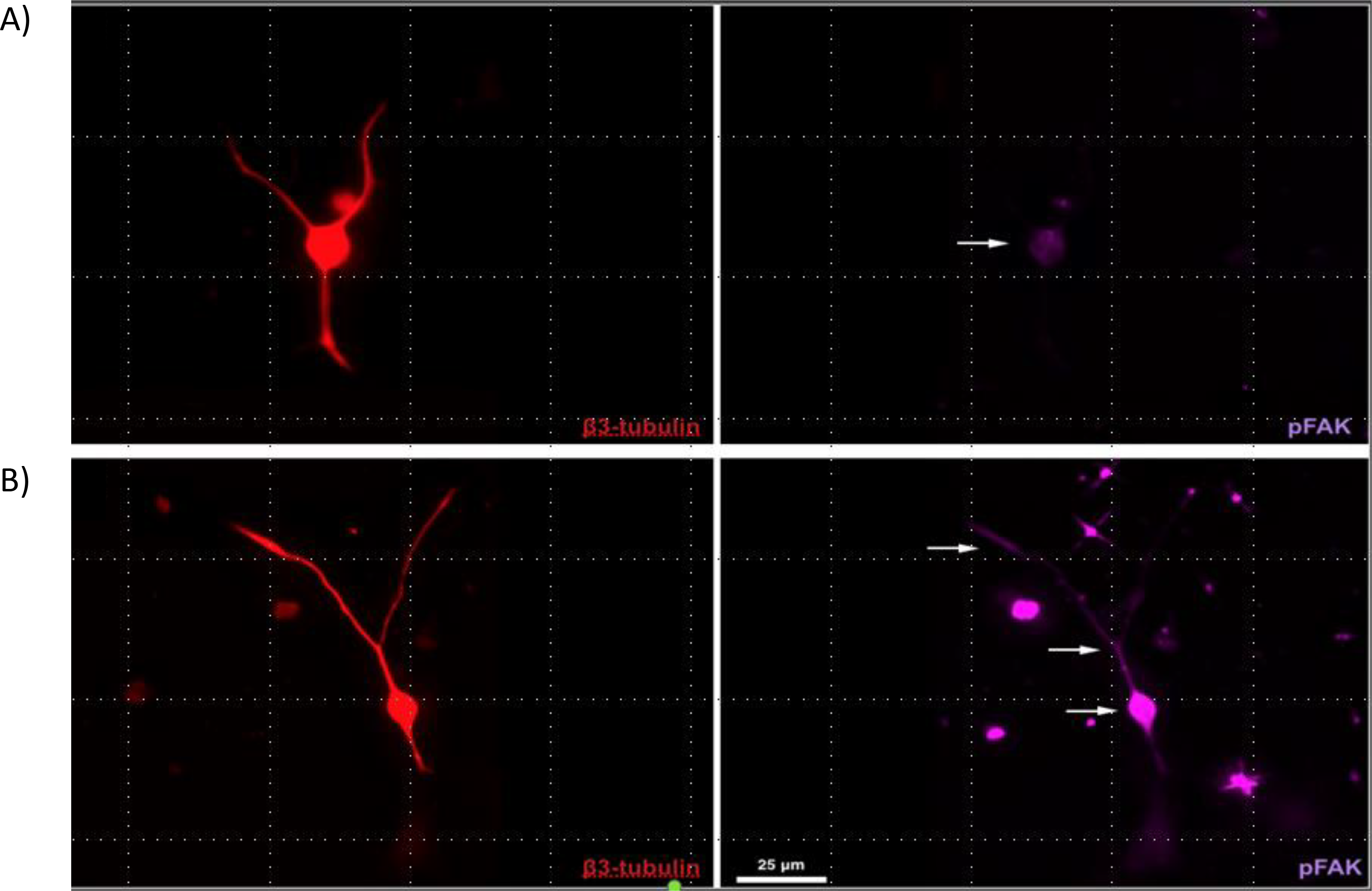

Supplement: S5 Fig — DRGs plated onto CSA and transfected with targeted chABC (Bottom panels, B) or GFP (Top panels, A) and stained for β-tubulin (Left hand panels) or pFAK (Right hand panels). Left hand panel (Top) shows staining for β-tubulin III is present in both the cell body and the axons of controls, (A) and targeted ChABC transfected DRGs (bottom panel), B. Right hand panel (Top), A shows weak staining for pFAK in the cell body of GFP-transfected DRGs and no staining is observed in the axonal compartment. In contrast, targeted ChABC transfected DRGs show bright staining for pFAK in the cell bodies and diffuse staining in the axonal compartment, indicating β-integrin activation, (bottom right hand panel, B). (TIF) [file pone.0221851.s006.tif]

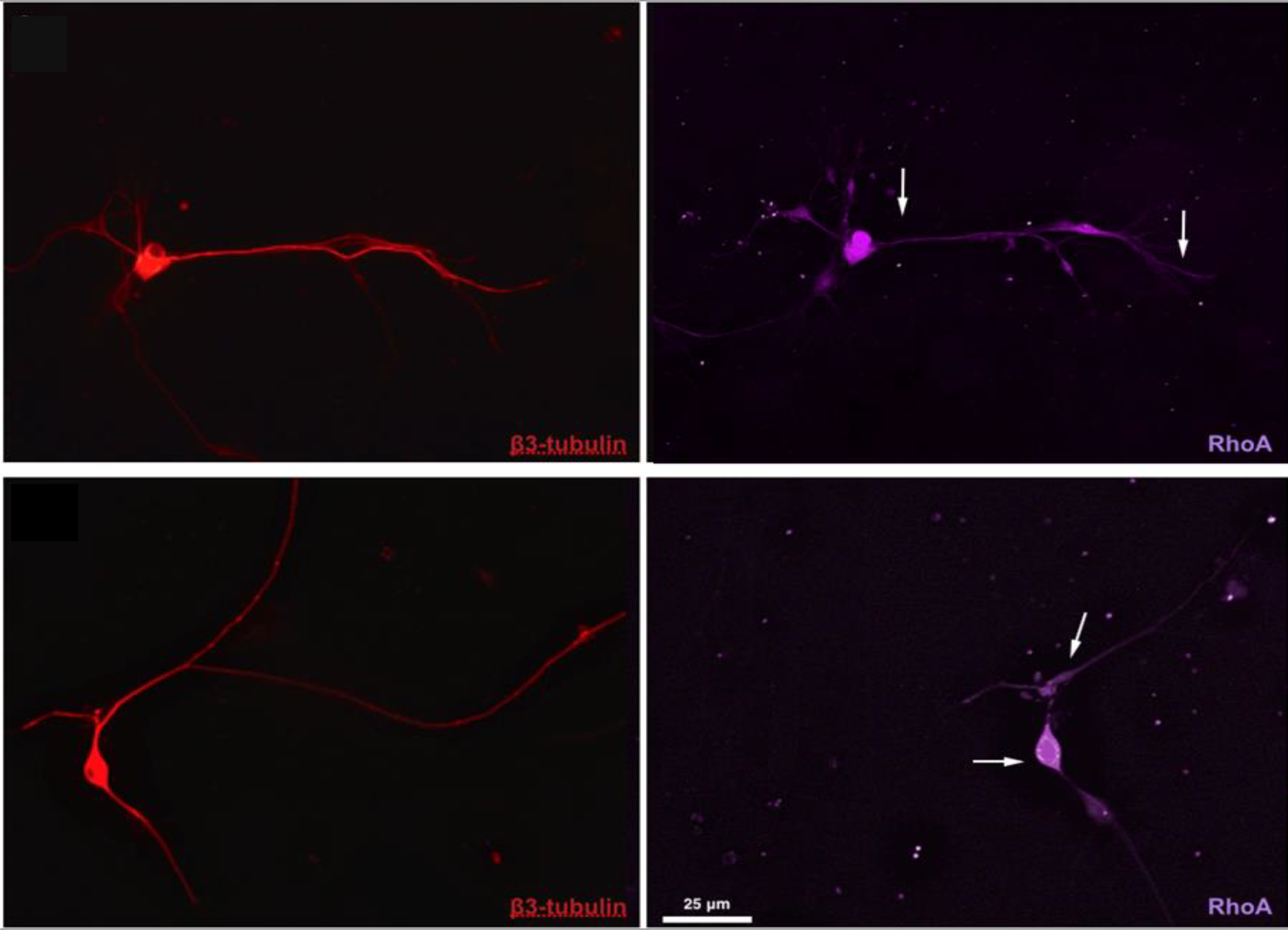

Supplement: S6 Fig — Rho A-staining of DRGs plated onto CSA and transfected with targeted chABC (bottom panel) or GFP (Top panel). Left hand panels show staining for β-tubulin III is present in both the cell body and the axons of controls (Top) and DRGs transfected with targeted ChABC(bottom). Right hand panels show strong staining for RhoA in both the cell body and axons of control neurons (Top). Neurons transfected with targeted ChABC show staining for RhoA in the cell body, but weak staining of the axonal compartment (bottom). (TIF) [file pone.0221851.s007.tif]
